# Serum fatty acid profiles in systemic lupus erythematosus and patient reported outcomes: The Michigan Lupus Epidemiology & Surveillance (MILES) Program

**DOI:** 10.3389/fimmu.2024.1459297

**Published:** 2024-12-18

**Authors:** Kristen N. Gilley, Jenifer I. Fenton, Suzanna M. Zick, Kexin Li, Lu Wang, Wendy Marder, W. Joseph McCune, Raghav Jain, Sidney Herndon-Fenton, Afton L. Hassett, Kamil E. Barbour, James J. Pestka, Emily C. Somers

**Affiliations:** ^1^ University of Michigan, Department of Internal Medicine, Ann Arbor, MI, United States; ^2^ University of Michigan, Department of Epidemiology, Ann Arbor, MI, United States; ^3^ Michigan State University, Department of Food Science and Human Nutrition, East Lansing, MI, United States; ^4^ University of Michigan, Department of Family Medicine, Ann Arbor, MI, United States; ^5^ University of Michigan, Department of Biostatistics, Ann Arbor, MI, United States; ^6^ University of Michigan, Department of Obstetrics & Gynecology, Ann Arbor, MI, United States; ^7^ University of Michigan, Department of Anesthesiology, Ann Arbor, MI, United States; ^8^ Centers for Disease Control and Prevention, Division of Population Health, National Center for Chronic Disease Prevention and Health Promotion, Atlanta, GA, United States; ^9^ Michigan State University, Department of Microbiology, Genetics and Immunology, East Lansing, MI, United States; ^10^ Michigan State University, Institute for Integrative Toxicology, East Lansing, MI, United States; ^11^ University of Michigan, Department of Environmental Health Sciences, Ann Arbor, MI, United States

**Keywords:** autoimmune, omega-3 fatty acid, polyunsaturated fatty acid (PUFA), docosahexaenoic acid (DHA), saturated fatty acid (SFA), monounsaturated fatty acid (MUFA), pain, sleep

## Abstract

**Introduction:**

Despite progress in systemic lupus erythematosus (SLE) treatment, challenges persist in medication adherence due to side effects and costs. Precision nutrition, particularly adjusting fatty acid intake, offers a cost-effective strategy for enhancing SLE management. Prior research, including our own, indicates that increased consumption of omega-3 polyunsaturated fatty acids (PUFAs) correlates with improved outcomes in SLE patients. Here we build upon these findings by investigating associations between serum fatty acids—grouped as PUFAs, monounsaturated fatty acids (MUFAs), and saturated fatty acids (SFAs)—and lupus activity, pain, and sleep disturbance.

**Methods:**

Using data from 418 participants with SLE in the Michigan Lupus Epidemiology and Surveillance (MILES) Cohort, we examined associations between serum levels of 25 fatty acids determined by GC-MS and patient-reported outcomes. Disease activity, pain, and sleep quality were assessed using standardized questionnaires. Generalized additive models and partial residual plots were utilized to examine the linearity of fatty acid effects. Variable selection was performed using Least Absolute Shrinkage and Selection Operator (LASSO), followed by multiple linear regression adjusting for sociodemographic factors.

**Results:**

Findings indicated favorable associations between ω-3 PUFAs—and, to a lesser extent, ω-6 PUFAs—and patient-reported outcomes, while MUFAs and SFAs showed unfavorable associations. Docosahexaenoic acid (DHA), an omega-3 PUFA, exhibited the most robust favorable associations across all outcomes. Additionally, the omega-3 α-linolenic acid (ALA) was linked to reduced pain, whereas eicosapentaenoic acid (EPA), another omega-3, was associated with worsened disease activity and pain. Among omega-6 PUFAs, dihomo-γ-linolenic acid (DGLA) was favorably associated with disease activity, while the omega-9 PUFA Mead acid was linked to increased pain.

**Discussion:**

These findings underscore the prospect that increased tissue levels of long-chain omega-3 PUFAs, particularly DHA, are favorably associated with SLE outcomes. Although further research is needed to establish causal relationships, existing evidence supports the role of omega-3 PUFAs in managing cardiovascular and chronic kidney disease, common SLE comorbidities. Most study participants exhibited low omega-3 PUFA status, suggesting substantial potential for improvement through targeted dietary interventions and supplementation. This study supports a potential role for precision nutrition in comprehensive SLE management, considering the impact of PUFAs, SFAs and MUFAs.

## 1 Introduction

Systemic lupus erythematosus (SLE or lupus) is an autoimmune disease characterized by fluctuating levels of disease activity or “flares” which can result in serious morbidities and irreversible organ damage. Over the last few decades, evolving therapeutic strategies for SLE have improved survival (1, 2). However mainstay treatments, which include corticosteroids, immunosuppressives, and biologics, are associated with substantial toxicities (3–5) and often do not adequately address issues, such as chronic pain and profound fatigue, that frequently impact the lives of persons with lupus. Moreover, one in five persons with SLE report non-adherence to prescription medications due to costs, and a similar fraction have sought lower-cost treatment alternatives from their providers (6, 7). In 2020, the National Institutes of Health unveiled a decade-long strategic blueprint centered on precision nutrition, with a primary goal of developing evidence-based interventions for reducing the burden of disease in clinical settings (8). While dietary interventions hold promise as part of comprehensive disease management, the majority of persons with SLE report a lack of dietary discussions with their doctor (9).

One of the most intriguing precision nutrition strategies proposes alteration of the cellular lipidome by modifying fatty acid consumption patterns, as fatty acids play a role in both pro- and anti-inflammatory pathways (10). Multiple studies have detailed the role of omega-6 (ω-6) polyunsaturated fatty acids (PUFAs), which include linoleic acid (LA) and its elongation product arachidonic acid (AA), in inflammation and pain in rheumatic diseases (11–13). Other research has explored AA metabolism and identified potential therapeutic targets for decreasing inflammation and pain (14). Importantly, ω-3 PUFAs can interfere with the above AA-driven proinflammatory pathways (10) and have been proposed as non-steroidal treatments in human discogenic pain, neuropathic pain, and inflammatory joint pain (15–17). LA is found in plant oils (*e.g.*, soybean, corn, and peanut) and considered to be an essential FA (18). The ω-3 PUFA α-linolenic acid (ALA), another essential FA, is found in certain nuts, seeds, and seed oils, while eicosapentaenoic acid (EPA) and docosahexaenoic acid (DHA), products of ALA elongation by certain microalgae, are bioconcentrated in oily fish such as salmon and herring. Because of intensive agricultural practices, LA is consumed to excess in the Western diet, skewing the proportion of ω-6 PUFAs consumed relative to ω-3 PUFAs. Reversing this skewed pattern by increasing ω-3 PUFA intake via diet or supplements may hold promise as an intervention against inflammatory and autoimmune diseases.

The effects of fish oil supplements on rheumatoid arthritis (RA) and other rheumatic diseases have been studied over the last four decades. A systematic review and meta-analysis of 20 randomized controlled trials found that ω-3 PUFA supplementation significantly improved several RA disease-activity markers, including a self-reported pain scale (19). A subsequent, more-encompassing meta-analysis of 30 randomized controlled clinical trials in patients with inflammatory rheumatic diseases (RA, ankylosing spondylitis, psoriatic arthritis) reported improved disease activity scores in individuals consuming PUFAs, especially ω-3s, exceeding 2 g/d for 3 to 6 months (20). In contrast, a 2024 meta-analysis of 23 randomized placebo-controlled trials reported limited benefits of ω-3 PUFA supplementation in RA patients; however, it also highlighted critical methodological pitfalls in evaluated trials (21). Other fatty acids besides PUFAs, such as monounsaturated fatty acids (MUFAs) (22) and saturated fatty acids (SFAs) (23), have the potential to impact RA and other rheumatic diseases, yet remain understudied despite being frequently mentioned in dietary guidance for patients with these diseases (24). Dietary fats and oils have a mix of fatty acid types. Those predominant in MUFAs include olive and avocado oils, while those rich in SFAs include coconut and palm oils, lard, red meats, and whole-fat dairy products. Ultra-processed foods, which represent a large portion of the American diet, contain considerable MUFAs, mainly from high-oleic acid varieties of soybean and sunflower oils, and SFAs (25).

Clinical research on fatty acids in SLE is comparatively less developed than for RA (26). Our group has previously reported that dietary intake levels of PUFAs, as measured by food frequency questionnaire, were associated with patient-reported outcomes in SLE. Specifically, a higher intake of ω-3 PUFAs and lower ratio of ω-6 to ω-3 PUFAs were found to be beneficial (27). Consistent with our findings, a subsequent German study also employing dietary history reported that increased dietary ω-3 PUFA consumption from fish was linked to lower SLE disease activity (28). In this investigation, we extend our work by quantifying a panel of serum fatty acids in a population-based cohort of persons with SLE to provide more detailed characterization of lipidome profiles. Here we report on associations between serum fatty acids, as groups (*e.g.*, PUFA, MUFA, SFA) and individually, and their associations with patient-reported outcomes of lupus activity, pain, and sleep disturbance in SLE.

## 2 Methods

### 2.1 Study Population

This study is based on the Michigan Lupus Epidemiology and Surveillance (MILES) Cohort, comprised of adults with lupus recruited from the MILES Surveillance Registry in southeastern Michigan, described elsewhere (29, 30). Institutional Review Board (IRB) approval was obtained from both the University of Michigan and the Michigan Department of Health and Human Services. All cohort participants provided written, informed consent.

### 2.2 Data collection

Data from the baseline cohort visit in 2014–2015 were utilized for this study. Sociodemographic information was collected by structured interview and questionnaires with items modeled after those used in the National Health and Nutrition Examination Survey (31). Race and ethnicity were self-reported. Physical measures included weight and height measured at the study visit, with body mass index calculated as kg/m^2^. Health insurance coverage in the preceding 12 months was categorized as public (Medicare and/or Medicaid), private or non-public, and none.

### 2.3 Patient reported outcome measures

Patient-reported outcome (PRO) measures were collected and scored according to each instrument’s instructions. The Systemic Lupus Activity Questionnaire (SLAQ) (32), which is validated for epidemiologic studies, was used to measure lupus disease activity during the past 30 days. It is comprised of 24 items (score range 0–47); a higher score indicates greater (worse) levels of disease activity. Pain was assessed using the bodily pain subscale from the RAND Medical Outcomes Study 36-item Short-Form-Survey (SF-36) instrument (33); scores ranged from 0–100, with higher scores representing better health for the domain being measured. Sleep quality was measured using the Patient-Reported Outcomes Measurement Information System (PROMIS) Sleep Disturbance Short Form 8b, version 1.0 (34), which assesses perceptions of sleep quality, depth and restoration. Higher T-scores represent worse sleep quality.

### 2.4 Quantification of serum fatty acids

Non-fasting blood was collected by standard venipuncture at the same study visit as the questionnaire and interview data. Coagulant-free red top vacutainers were used for serum specimens, which were processed and aliquoted using standardized protocols and stored at -70°C. Aliquots were rapidly thawed and analyzed by gas chromatography-mass spectrometry (GC-MS) to determine concentrations of the fatty acids indicated in **Table 1**.

**Table 1:** Serum fatty acid levels in study participants with lupus (n=418).

	Carbon:Bond ^a^	Concentration (μg/mL) mean (SD)	Percentage of total FA % (SD)
Polyunsaturated Fatty Acids (PUFA) – total		1569.7 (574.0)	44.1 (5.4)
**ω-3 PUFA – total**		**132.9 (46.0)**	**3.8 (0.8)**
α-linolenic (ALA)	18:3 (ω-3)	24.3 (13.1)	0.7 (0.2)
Eicosapentaenoic (EPA)	20:5 (ω-3)	44.0 (18.5)	1.2 (0.4)
Docosapentaenoic (ω-3 DPA)	22:5 (ω-3)	22.3 (4.9)	0.7 (0.2)
Docosahexaenoic (DHA)	22:6 (ω-3)	42.4 (24.0)	1.2 (0.6)
**ω-6 PUFA – total**		**1433.6 (546.0)**	**40.2 (5.3)**
Linoleic (LA)	18:2 (ω-6)	1065.3 (453.2)	29.7 (4.8)
Linoelaidic	18:2 (ω-6)*t*	7.6 (2.2)	0.2 (0.1)
γ-Linolenic (GLA)	18:3 (ω-6)	18.7 (12.2)	0.5 (0.2)
Eicosadienoic	20:2 (ω-6)	6.5 (1.4)	0.2 (0.1)
Dihomo-γ-linolenic (DGLA)	20:3 (ω-6)	20.9 (10.0)	0.6 (0.2)
Arachidonic (AA)	20:4 (ω-6)	279.3 (108.6)	7.9 (2.1)
Docosatetraenoic	22:4 (ω-6)	17.9 (6.2)	0.5 (0.1)
Docosapentaenoic (ω-6 DPA)	22:5 (ω-6)	17.5 (3.7)	0.5 (0.1)
**ω-9 PUFA – total**		**3.2 (7.0)**	**0.1 (0.2)**
Mead	20:3 (ω-9)	3.2 (7.0)	0.1 (0.2)
Monounsaturated Fatty Acids (MUFA) – total		841.5 (441.8)	22.9 (4.1)
Palmitoleic	16:1 (ω-7)	63.6 (56.3)	1.7 (1.0)
Palmitelaidic	16:1 (ω-7)*t*	11.5 (5.5)	0.3 (0.1)
Oleic	18:1 (ω-9)	718.8 (388.7)	19.5 (3.7)
Elaidic	18:1 (ω-9)*t*	15.8 (4.7)	0.5 (0.1)
Eicosenoic	20:1 (ω-9)	8.2 (1.2)	0.2 (0.1)
Nervonic	24:1 (ω-9)	23.6 (6.5)	0.7 (0.2)
Saturated Fatty Acids (SFA) – total		1206.7 (556.3)	33.0 (2.6)
Myristic	14:0	38.3 (31.5)	1.0 (0.5)
Palmitic	16:0	834.4 (409.4)	22.7 (2.3)
Stearic	18:0	282.1 (125.6)	7.8 (1.0)
Arachidic	20:0	11.1 (3.0)	0.3 (0.1)
Behenic	22:0	23.7 (6.3)	0.7 (0.2)
Lignoceric	24:0	17.0 (4.4)	0.5 (0.2)

Sorted by major fatty acid group [PUFA (ω-3, ω-6, ω-9), MUFA, SFA] and carbon number: bond. Data are presented as concentration (μg/mL) and percentage of total fatty acids.

FA, fatty acid; MUFA, monounsaturated FA; PUFA, polyunsaturated FA; SFA, saturated FA. Bold indicates pooled fatty acid group values.

a omega nomenclature is C:B (ω-*x*) where C=number of carbons, B=number of carbon-carbon double bonds, and (*x*)=position of first double bond from the methyl (ω) end. *t* after (ω-*x*) indicates *trans* fatty acid.

#### 2.4.1 Reagents and standards

All reagents were analytical grade and purchased from Sigma-Aldrich (St. Louis, MO, USA). Stearic acid-d_35_ was used as an internal standard (Sigma-Aldrich; St. Louis, MO). Palmitelaidic, eicosatrienoic acid, docosatetraenoic, ω-6 docosapentaenoic (DPA ω-6) and ω-3 docosapentaenoic (DPA ω-3) acid methyl ester standards were purchased from Cayman Chemical (Ann Arbor, MI, USA). All other fatty acid methyl ester (FAME) standard curves were created using Supelco 37 Component FAME Mix (Sigma-Aldrich; St. Louis, MO).

#### 2.4.2 Fatty acid extraction and derivatization

Total fatty acids (FAs) were extracted and methylated using the one-step transesterification method of Lepage and Roy (35) as modified for large clinical studies (36). Briefly, 2 mL of a 1.8:0.2 (v/v) methanol:acetyl chloride solution with 0.01% (w/v) butylated hydroxy-toluene and internal standard was added to 100 μL of serum in a test tube. The mixture was heated for 1h at 100°C, cooled to room temperature, then neutralized with 2 mL of 5% (w/v) sodium bicarbonate solution. FAMEs were extracted twice with 2 mL hexane into a new test tube, dried down under nitrogen, resuspended in isooctane, and transferred to GC vials. Samples were stored at -20°C until GC-MS analysis.

#### 2.4.3 Gas chromatography-mass spectrometry analysis

The Perkin Elmer (Waltham, MA, USA) 680/600S GC-MS with an Agilent Technologies (Santa Clara, CA, USA) DB-23, 30-m column was used for FAME quantification. The GC temperature parameters were as follows: initial temperature at 100°C for 0.5 minutes; ramped 8.0 degrees per minute to 200°C, then 2.5 degrees per minute to 220°C, and finally 10 degrees per minute to 240°C, where it was held for 2 minutes. Seven-point calibration curves were created using purchased FAME standards and selective-ion monitoring was used to quantify FAMEs. GC-MS data analysis was conducted using TargetLynx v4.0 (Waters Corporation; Milford, MA, USA). FAME values were normalized per mL of serum and expressed as percent of total FA.

### 2.5 Statistical analysis

Serum fatty acid concentrations (μg/mL) below the limit of detection (LOD) were imputed as LOD/√2. Mean and standard deviation were computed for continuous variables, and frequency and percentage for categorical variables. P-values of <0.05 were considered statistically significant. For each fatty acid group (*e.g.*, ω-3 PUFA, ω-6 PUFA, MUFA, SFA), the total level was computed as the sum of the concentrations of individual fatty acids within the group. Since the ω-9 PUFA category was comprised of a single FA measured (Mead), it was not included in group-level analyses. Individual and group fatty acid levels were expressed as a percentage of total fatty acids (%FA) by dividing the concentration of the respective fatty acid or group by the total fatty acid level (*i.e.*, the sum of all fatty acids measured), then multiplying by 100. An erythrocyte membrane-based Omega-3 Index (O3I), calculated as the sum of EPA and DHA as %FA in red blood cells, has been proposed (37), with the relationship to plasma levels published elsewhere (38). Applying this equation, the estimated O3I (eO3I) in this study was calculated as follows: (*serum EPA* %*FA* + *DHA* %*FA* + 0.0141)/0.7799. Absolute and relative levels of major fatty acids from matched human serum and plasma samples are highly comparable (39). Using classification from Schuchardt et al. (40) based on O3I of 167,347 persons in seven countries, we categorized the eO3I as follows: desirable (≥8%), moderate (≥6% to 8%), low (≥4% to 6%), and very low (<4%).

To assess the potential non-linear relationships between outcomes and the fatty acid groups, generalized additive models were used along with partial residual plots, which graphed the residuals from the model against each predictor while controlling for other group variables. Serum concentrations rather than %FA were used in the models with more than one fatty acid group to avoid multicollinearity. For the 25 individual fatty acids, variable selection was performed using Least Absolute Shrinkage and Selection Operator (LASSO) (41), with standardized values of the serum concentrations and performed separately for each of the outcomes. This approach applies an L1-norm regularization penalty to produce sparce coefficients and effectively identify relevant features associated with the outcomes. Regularization paths were used to visualize how different penalties shrink the coefficients. The selected variables were used for post-LASSO regression modeling to examine fatty acids (groups or the LASSO-selected individual FAs) in association with patient-reported outcomes. For the multivariable linear regression models, primary analyses adjusted for sex, age, BMI, and race. Secondary analyses additionally adjusted for health insurance coverage [private, public (Medicaid/Medicare), or none] and household income above or below the US median. Statistical analyses were performed using Stata v.18 and R version 4.3.2 software. For data visualizations, the colorblind accessible palette by Okabe and Ito was used (42).

## 3 Results

### 3.1 Characteristics of study population

This study included 418 participants with lupus from the MILES Cohort, with available serum samples from the baseline visit for the fatty acid laboratory assays. Demographic characteristics are summarized in **Table 2**. The average age was approximately 54 years, the majority of participants were female (93%), and most participants self-reported their race as White (54%) or Black (42%). Among the participants, 114 (27.3%) reported regular intake of ω-3 supplements (fish and/or flaxseed oil) more than once per week.

**Table 2:** Characteristics of study participants with lupus.

Characteristic	SLE (n=418)
**Age (years)**	53.5 ± 12.3
Sex	
Female	387 (92.6%)
Male	31 (7.4%)
Race	
White	224 (53.6%)
Black	174 (41.6%)
Other	20 (4.8%)
**Hispanic ethnicity**	17 (4.1%)
**Arab North African ancestry**	10 (2.4%)
Health insurance coverage	
Private/other	197 (47.1%)
Public (Medicaid, Medicare)	213 (51.0%)
None	8 (1.9%)
**Income below US median**	212 (50.7%)
Education	
Less than HS	36 (8.6%)
High School/GED	42 (10.0%)
Some College/Associate’s	177 (42.3%)
Bachelor’s	83 (19.9%)
Graduate/Professional	79 (18.9%)
Undisclosed	1 (0.2%)
**BMI (kg/m^2^)**	30.0 ± 7.9
Patient-reported outcomes	
SLAQ	12.7 (7.9)
SF-36 Pain	53.0 (26.9)
PROMIS Sleep Disturbance	56.3 (8.8)

### 3.2 Serum fatty acid levels in SLE population

Serum levels for individual fatty acids and groups are summarized in **Table 1**. ω-6 PUFAs and SFAs were predominant, representing 40% and 33% of total fatty acids, respectively. The average eO3I was 3.2% (SD 0.9), with most participants (84.2%) having a “very low” eO3I of <4%, followed by 15.3% “low”, 0.5% “moderate” and 0% “desirable.”

### 3.3 Fatty acid groups and patient-reported outcomes

**Figure 1:**
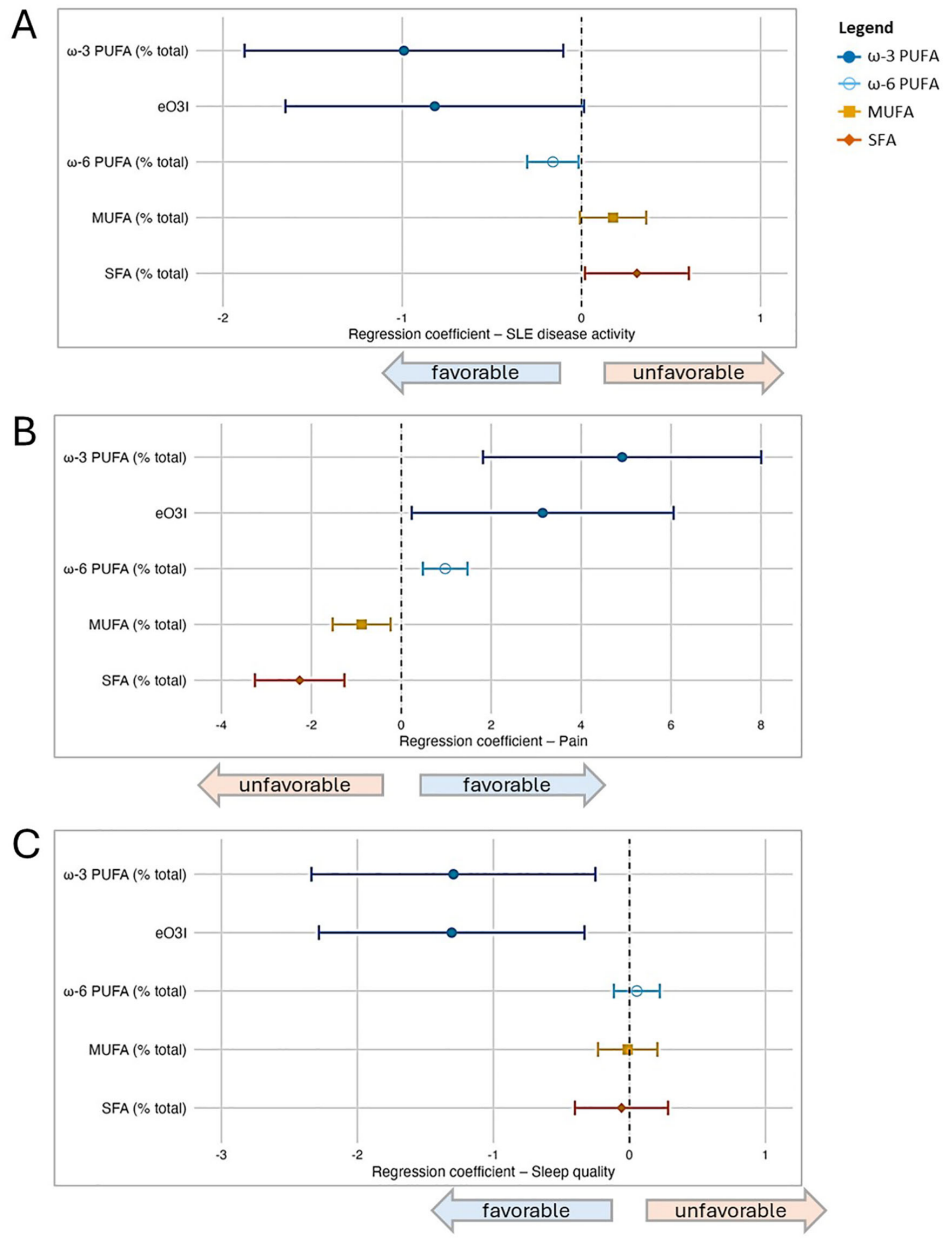
Associations between fatty acid groupings and patient-reported outcomes (PROs) in SLE based on a series of multivariable regression models. In addition to the four major fatty acid groups under study (ω-3 PUFA, ω-6 PUFA, MUFA, SFA), the estimated Omega-3 Index (eO3I) was also included; all were expressed as percentage of total fatty acids in this part of the analysis. Separate multivariable regression models were performed for each fatty acid group and outcome pair (due to multicollinearity between groups), and all models were adjusted for covariates (sex, age, BMI, and race). In the forest plots, symbols designate regression coefficients (β) and horizontal lines designate 95% confidence intervals (CIs). Estimates where the 95% CIs do not overlap 0 are considered statistically significant at the 0.05 level. For the SLAQ and sleep outcomes, higher scores represent worse disease activity and sleep quality (*i.e.*, negative associations are favorable). For the pain outcome, higher scores represent better health in that domain (*i.e.*, positive associations are favorable). Symbol marker colors/shapes correspond to fatty groups: dark blue/solid circle=ω-3 PUFA; light blue/hollow circle=ω-6 PUFA, orange/solid square=MUFA, red-orange/solid diamond=SFA. FA, fatty acid; MUFA, monounsaturated FA; PUFA, polyunsaturated FA; SFA, saturated FA. **(A)** Lupus disease activity (SLAQ). **(B)** Pain (SF-36). **(C)** Sleep quality (PROMIS Sleep Disturbance).

When assessing four fatty acid groups (ω-3 PUFA, ω-6 PUFA, MUFA, SFA) and eO3I expressed as percentage of total FA, in separate multivariable models adjusted for the primary model covariates (sex, age, BMI, and race) (**Figure 1**), we found that PUFAs (ω-3, eO3I, ω-6) were favorably associated with disease activity and pain, whereas MUFAs and SFAs were unfavorably associated. For the sleep quality outcome, ω-3 PUFAs (including as represented by eO3I) were also favorably associated, while associations were not detected for the other fatty acid groups.

We then modeled the four fatty acid group serum concentrations simultaneously (each controlled for the effects of the other three groups, as well as other covariates) in separate multivariable models for each outcome (**Table 3**). When evidence of non-linearity was observed, the corresponding fatty acid group segments were utilized. For disease activity, the ω-3 PUFA group remained favorably associated when controlling for the other fatty acid groups and primary model covariates: for each 1 μg/mL increase of ω-3 concentration, there was a reduction in SLAQ disease activity score of 0.03 [β -0.03 (95% CI -0.05, -0.00)]. The ω-6 PUFA group, within the segment of >1900 μg/mL, trended towards a favorable association of weaker magnitude than ω-3 PUFAs [β -0.003 (95% CI -0.01, 0.00)]. In secondary models that additionally adjusted for health insurance and income, the effect estimates remained similar but did not reach significance.

**Table 3:** Associations between fatty acid groups and patient-reported outcomes (PROs) in persons with lupus.

	SLAQ ^a^ β coefficient (95% CI) ^c^	Pain ^b^ β coefficient (95% CI)	Sleep quality ^a^ β coefficient (95% CI)
Age (years)	0.008 (-0.053, 0.068)	-0.051 (-0.257, 0.154)	0.004 (-0.067, 0.075)
Male sex (Female referent)	-4.142 (-6.915, -1.370)**	1.107 (-8.325, 10.539)	-2.467 (-5.793, 0.858)
BMI (kg/m^2^)	0.178 (0.084, 0.273)***	-0.582 (-0.903, -0.261)***	0.123 (0.011, 0.235)*
Race (White referent)			
Black	1.799 (0.239, 3.358)	-6.889 (-12.223, -1.556)*	0.448 (-1.396, 2.292)
Other/unknown	6.081 (2.519, 9.644)***	-2.380 (-14.494, 9.734)	2.507 (-1.706, 6.719)
FA group concentrations			
ω-3 PUFA (μg/mL)	-0.026 (-0.051, -0.001)*	0.100 (0.018, 0.183)*	-0.026 (-0.055, 0.004)†
ω-6 PUFA, segment 1 (μg/mL) ^d^	0.000 (-0.003, 0.004)	0.016 (0.006, 0.025)***	0.004 (-0.001, 0.008)†
ω-6 PUFA, segment 2 (μg/mL) ^d^	-0.003 (-0.006, 0.000)†	NA	0.000 (-0.003, 0.004)
MUFA segment 1 (μg/mL) ^e^	0.002 (-0.003, 0.006)	-0.031 (-0.059, -0.004)*	0.002 (-0.004, 0.007)
MUFA segment 2 (μg/mL) ^e^	NA	0.015 (-0.002, 0.031)†	NA
SFA (μg/mL)	0.002 (-0.003, 0.007)	-0.032 (-0.048, -0.015)***	-0.001 (-0.007, 0.005)

Results based on multivariable linear regression with concentrations (μg/mL) for the four fatty acid groups simultaneously modeled, along with primary model covariates (age, sex BMI, race). Separate models were conducted for each outcome.

BMI, body mass index; FA, fatty acid; MUFA, monounsaturated FA; NA, not applicable; PUFA, polyunsaturated FA; SFA, saturated FA; SLAQ, Systemic Lupus Activity Questionnaire.

a Higher score worse (SLAQ, sleep).

b Higher score better (pain).

c Significance levels indicated to right of 95% CIs as follows: [***]p<0.001; [**]p<0.01; [*]p<0.05; [†]p<0.1.

d ω-6 segments as applicable for non-linear associations: SLAQ ≤1900 and >1900 μg/mL; Sleep ≤1650 and >1650 μg/mL. NA indicates that non-linearity was not detected, therefore segment 1 represents the entire range.

e MUFA segments as applicable for non-linear associations: Pain ≤750 and >750 μg/mL. NA indicates that non-linearity was not detected, therefore segment 1 represents the entire range.

For the pain outcome, PUFAs were favorably associated, with the ω-3 PUFAs having a stronger magnitude of association than ω-6 PUFAs [ω-3 β 0.10 (95% CI 0.02, 0.18); ω-6 β 0.02 (95% CI 0.01, 0.03)]. In contrast, MUFAs within the segment ≤750 μg/mL and SFAs were unfavorably associated with pain [MUFA ≤750 μg/mL β -0.03 (95% CI -0.06, -0.00); SFA β -0.03 (95% CI -0.05, -0.02)]. In secondary models, the ω-3 PUFA association did not reach significance, whereas results for the other three fatty acid groups remained significant. For sleep quality, in the primary analysis there was a trend towards a favorable association for ω-3 [β -0.03 (95% CI -0.06, 0.00] and unfavorable association for the lower segment of ω-6 ≤1650 μg/mL [β 0.004 (95% CI -0.001, 0.008)]. Directions of association were similar in secondary models.

### 3.4 Individual fatty acids and patient-reported outcomes

**Figure 2:**
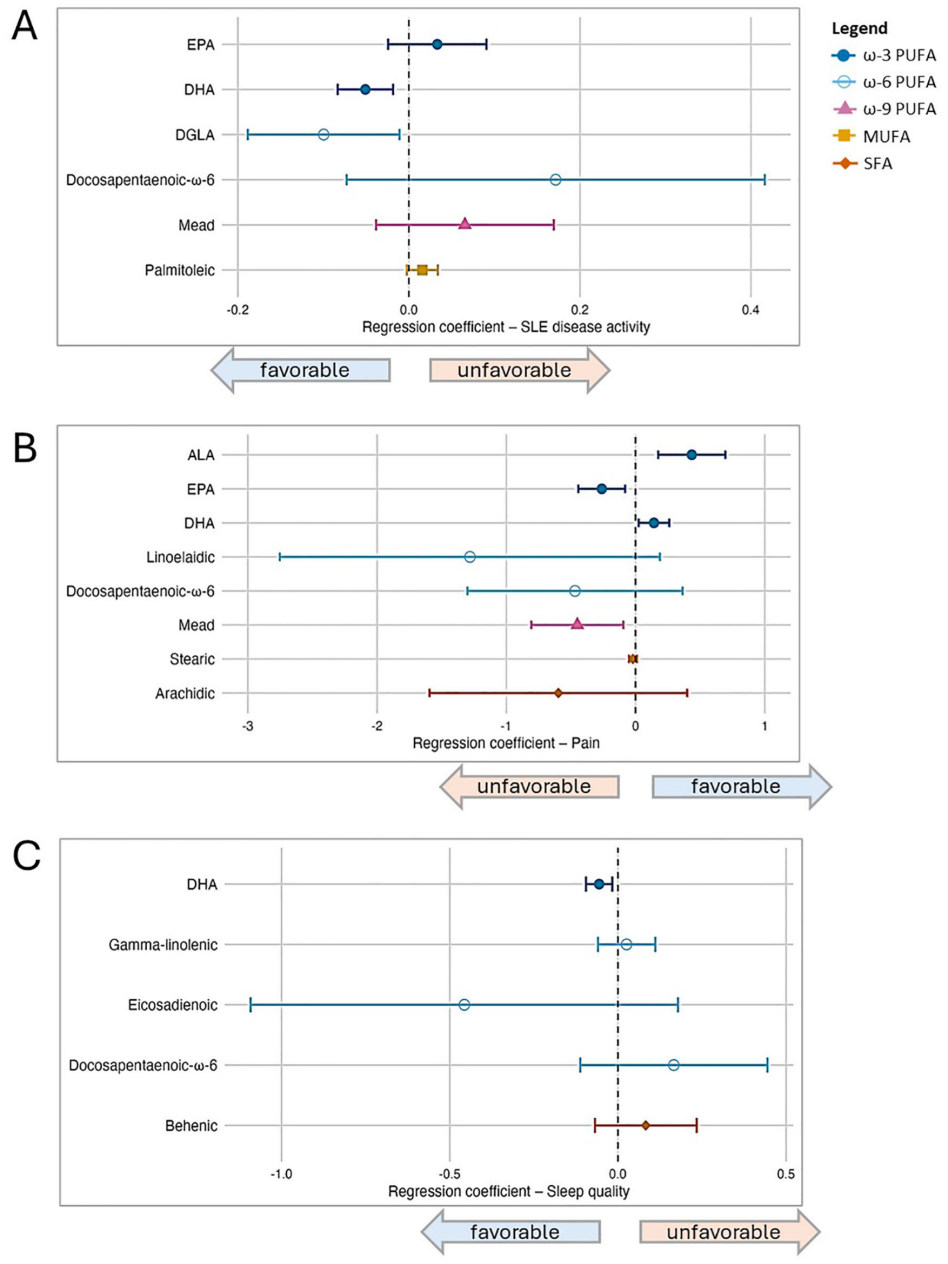
Associations between individual fatty acid serum concentrations (μg/mL) and patient-reported outcomes in SLE, based on post-LASSO multivariable regression. Separate models are presented according to outcome, and each model included the set of fatty acids selected by Least Absolute Shrinkage and Selection Operator (LASSO), as well as covariates (sex, age, BMI, and race). In the forest plots, symbols designate regression coefficients (β) and horizontal lines designate 95% confidence intervals (CIs). Estimates where the 95% CIs do not overlap 0 are considered statistically significant at the 0.05 level. For the SLAQ and sleep outcomes, higher scores represent worse disease activity and sleep quality (*i.e.*, negative associations are favorable). For the pain outcome, higher scores represent better health in that domain (*i.e.*, positive associations are favorable). Symbol marker colors/shapes correspond to fatty groups: dark blue/solid circle=ω-3 PUFA; light blue/hollow circle=ω-6 PUFA, purple/solid triangle=ω-9 PUFA, orange/solid square=MUFA, red-orange/solid diamond=SFA. FA, fatty acid; MUFA, monounsaturated FA; PUFA, polyunsaturated FA; SFA, saturated FA. **(A)** Lupus disease activity (SLAQ). **(B)** Pain (SF-36). **(C)** Sleep quality (PROMIS Sleep Disturbance).

Next, we modeled each of the 25 individual fatty acids separately and used LASSO regression to select the subset most relevant for each outcome. Forest plots from the post-LASSO multivariable models are displayed in **Figure 2**.

For disease activity, among six FAs selected by LASSO (listed in **Figure 2**), DHA (ω-3) and dihomo-γ-linolenic (DGLA; ω-6) were favorably associated and statistically significant when these six were modeled simultaneously, controlling for each other as well as other covariates: in the primary models, for each 1 μg/mL increase of DHA and DGLA, disease activity score decreased by 0.05 and 0.1 points, respectively [DHA β -0.05 (95% CI -0.08, -0.02); DGLA β -0.10 (95% CI -0.19, -0.01)]. EPA (ω-3) trended toward an unfavorable association [β 0.03 (95% CI -0.02, 0.09)]. Results in secondary models overall were similar, though the magnitude of association for EPA increased and reached statistical significance when both health insurance and income were added in the modeling, whereas the DGLA association was slightly attenuated.

For pain, eight individual FAs were selected by LASSO, among which in the primary models two were favorably associated [DHA (ω-3) β 0.14 (95% CI 0.02, 0.26); ALA (ω-3) β 0.44 (95% CI 0.18, 0.70)] and two unfavorably [EPA (ω-3) β -0.26 (95% CI -0.44, -0.08; Mead (ω-9) β -0.45 (95% CI -0.81, -0.09)]. There were no substantive changes in results from secondary models. For sleep quality, among five FAs selected by LASSO, DHA was favorably associated in multivariable modeling in the primary model [DHA β -0.06 (95% CI -0.10, -0.02)] as well as secondary models. No other individual FAs were significantly associated with sleep quality in the multivariable models.

## 4 Discussion

### 4.1 Summary of primary findings

In this study, we expanded upon our prior findings based on dietary history data that supported a beneficial role for ω-3 PUFA intake in SLE (27). Quantification of serum concentrations of 25 fatty acids representing four major fatty acid groups—ω-3 PUFAs, ω-6 PUFAs, MUFAs, and SFAs—provides a level of granularity beyond prior observational lupus studies in humans. In this population-based SLE cohort, when fatty acids were assessed as groups, associations with patient-reported outcomes of disease activity, pain, and sleep quality tended to be favorable for ω-3 PUFAs and, to a lesser extent, ω-6 PUFAs. Conversely, associations tended to be unfavorable for MUFAs and more so for SFAs. Among 25 individual FAs, the ω-3 fatty acid DHA emerged as having favorable associations across all three outcomes after accounting for the other fatty acids measured as well as sociodemographic factors. Multivariable analyses found another ω-3 PUFA, α-linolenic acid (ALA), was also favorably associated with pain. Unexpectedly, the ω-3 EPA was unfavorably associated with disease activity and pain when accounting for other fatty acids. Other than ω-3s, DGLA (ω-6) was favorably associated with disease activity, and Mead acid (ω-9) was unfavorably associated with pain.

### 4.2 Plausibility of PUFAs impacting SLE

The potential of ω-3 PUFAs to influence SLE disease activity is biologically plausible based on their well-established capacity to interfere with innate and adaptive immune cell activation via several mechanisms (43). DHA and EPA enhance membrane fluidity and disrupt lipid raft formation, thereby hindering the activation of transmembrane receptors associated with inflammatory signaling (44). In addition, both extracellular and intracellular phospholipases can cleave incorporated ω-3 PUFAs from the membrane (45, 46). Liberated ω-3 PUFAs can compete with ω-6 PUFAs as substrates for cyclooxygenases, lipoxygenases and cytochrome P450s thereby reducing the production of proinflammatory eicosanoids associated with rheumatic disease (12, 47). Released DHA and EPA may also activate transmembrane receptors or intracellular receptors linked to the suppression of proinflammatory signaling (48, 49). DHA undergoes metabolic processes to yield oxylipins that function as specialized pro-resolving mediators (SPMs) critical to resolving inflammation, such as maresins, resolvins, protectins, and anti-inflammatory epoxide metabolites (50–53). SPMs actively inhibit inflammatory signaling (54, 55) and promote the efferocytosis of dead cells (56, 57), both of which assume critical roles in arresting the pathogenesis of autoimmune diseases. Interestingly, as we expected, the ω-3 PUFA DHA was associated favorably with SLE disease activity; however, there was divergence for EPA. DHA and EPA are known to have different physiological effects (58, 59). While DHA has been reported to have higher efficacy against progression of lupus in mouse models (60), ours is the first report of such divergence in human SLE. This suggests that for SLE-specific outcomes, DHA may be a more important driver of benefits and that composite indicators of ω-3 PUFA status might obscure some clinical effects. Consistent with this notion, it is recognized that DHA has a broader impact than EPA on inflammatory markers (58, 61, 62). Lastly, it was interesting to note that DGLA was associated with favorable SLE outcomes. DGLA may exert anti-inflammatory effects via several mechanisms (63). This ω-6 PUFA can inhibit the production of inflammatory mediators such as ICAM-1, MCP-1, RANTES, and IL-6 (64). In addition, DGLA undergoes metabolism by cyclooxygenases to yield PGE1, a potent anti-inflammatory agent. DGLA can also be transformed into 15-HETrE by 15-lipoxygenase, which hampers the synthesis of AA-derived 5-lipoxygenase products such as leukotriene B4 (LTB4) (63). Consonant with DGLA’s anti-inflammatory actions, decreased circulating DGLA and DGLA/AA ratios are associated with total mortality in patients with acute cardiovascular disease (65).

### 4.3 Other clinical studies relating tissue PUFAs to SLE

Data supporting the benefits of increased ω-3 PUFA tissue status in relation to SLE clinical parameters are accruing. A study of 114 SLE patients reported that DHA and EPA in abdominal adipose tissue correlated negatively with disease activity, whereas there was a positive correlation with ω-6 PUFA status (66). However, no associations were found for adipose MUFA and SFA levels with SLE disease activity. An investigation of erythrocyte membrane fatty acids in 68 persons with SLE found that total ω-3 PUFAs were negatively associated with the systemic inflammatory marker C-reactive protein (CRP), while total ω-6 PUFAs were positively associated, although no association with SLE activity was detected (28). Finally, a recent pilot study of 30 SLE patients reported that serum EPA, DHA, and AA (ω-6) correlated inversely with anti-dsDNA antibody levels, while serum LA (ω-6) correlated positively with anti-nuclear antibody titers (67). However, there were no associations between these PUFAs and SLE disease activity.

### 4.4 PUFAs and pain

This is the first study in persons with SLE to show that serum ω-3 PUFAs as a group, and particularly ALA and DHA were associated favorably with pain. ω-3 PUFAs could potentially interfere with pain in several ways. Eicosanoids such as thromboxanes, prostaglandins, and leukotrienes generated by enzymatic metabolism of the ω-6 PUFAs AA and LA and released by immune cells or neurons, act as paracrine mediators by activating G-protein coupled receptors (GPCRs) and modulating ion channel activity in peripheral sensory neurons (68). These activations trigger second messenger signaling cascades that decrease the threshold of ion channels, including the transient receptor potential cation channel subfamily vanilloid 1 (TRPV1), leading to heightened activity of peripheral sensory neurons. ω-3 PUFAs can compete with ω-6 PUFAs as substrates for metabolic pathways, thereby reducing pain-associated eicosanoids and increasing the production and release of SPMs. SPMs have potent analgesic effects in rodent models of inflammatory, neuropathic, and cancer pain, acting through G protein-coupled receptors on immune cells, glial cells, and neurons; their absence impairs pain resolution (69). Interestingly, in our study, EPA was paradoxically associated with increased pain, which might reflect the lower potency of its SPMs relative to those of DHA (60). It was further notable that Mead acid levels were unfavorably associated with pain. Also known as 5,8,11-eicosatrienoic acid, Mead acid is an ω-9 PUFA formed through the elongation of oleic acid that has been reported to have implications in inflammation, cancer, dermatitis, and cystic fibrosis (70). Mead acid can be transformed into various specific proinflammatory lipid mediators via enzymatic pathways involving lipoxygenases and cyclooxygenases, such as hydroxyeicosatrienoic acids (HETrEs) and leukotrienes that potentially influence pain perception (71, 72). Altogether, our findings highlight the need for further investigation of how individual fatty acids influence SLE pain and their potential role as biomarkers for pain management strategies.

### 4.5 Association of SFAs and MUFAs with SLE activity and pain

We also found that increasing serum levels of SFAs and MUFAs were associated with worse SLE activity and pain outcomes. These pain associations remained significant when modeling the four fatty acid groups simultaneously. Research on the relationship of SFAs or MUFAs and SLE has been scant. Investigations in NZBWF1 mice reported more advanced lupus nephritis and shorter survival in mice fed SFA and MUFA diets as compared to ω-3 PUFA-rich diets (73, 74). Consistent with SFA-associated pain, a study in trauma-induced rats found that groups consuming increased long-chain SFAs had increased pain behavior (23). Tierney et al. reported that feeding mice diets containing high levels of SFA sensitized them to induce robust pain states to non-painful stimuli (75). In contrast with the observed non-linear association between the MUFA group and pain in our study, several preclinical studies have suggested intake of the MUFA oleic acid is associated with reduced neuropathic pain (76). A recent study in Michigan of ultra-processed ready-to-eat and fast foods revealed that SFAs and MUFAs accounted for approximately 37.9% and 38.6% of total dietary lipids, respectively, whereas ω-6 and ω-3 PUFAs accounted for 23.5% and 0.1%, respectively (25). Therefore, it might be speculated that consuming diets with whole foods rich in PUFAs, rather than ultra-processed foods, could simultaneously lower tissue SFAs and MUFAs, thereby improving SLE activity and pain.

### 4.6 ω-3 PUFAs and sleep quality

Our findings that ω-3 PUFAs as a group, and particularly DHA, were favorably associated with sleep quality in persons with SLE support prior findings from our group utilizing dietary history data (27). These results are largely compatible with a small body of literature in other populations. An RCT published in 2021 of healthy young adults in England with low oily fish intake found that daily supplementation for 26 weeks with DHA-rich (DHA 900 mg + EPA 270) but not EPA-rich (DHA 360 mg + EPA 900 mg) oil compared to placebo was associated with improvements in actigraphy-based measures of sleep quality, though benefits were not detected by subjective participant reported outcomes (77). An RCT published in 2022 in healthy Japanese adults (age ≥45 years) found that objectively measured sleep efficiency in the group with daily ω-3 supplementation for 12 weeks (DHA 576 mg + EPA 284 mg) was significantly improved compared to the placebo group (78). An NHANES-based study reported that levels of EPA, DHA, and total ω-3 (measured by EPA, DHA, and docosapentaenoic) were lower among those with very short sleep relative to normal sleep duration (79). A recent meta-analysis of 12 large studies from five countries reported that individuals with higher concentrations of ω-3 PUFAs were less likely to have sleep that exceeded the current recommended duration, suggestive of beneficial effects on sleep consolidation/quality (80). ω-3 fatty acids are involved in melatonin biosynthesis as they are needed for enzymatic reaction with arylalkylamine-N-acetyltransferase, suggesting a potential mechanism through which ω-3 may impact sleep (81).

### 4.7 Relation of eO3I to SLE, pain, and sleep quality

The O3I is a widely used biomarker of ω-3 PUFA status determinable by a simple blood test (37). A “desirable” O3I target value of at least 8% has been proposed based on cardiovascular and longevity studies (40). Typical O3I levels in the US fall in the “low” range of 4 to 6% (82). The mean eO3I of 3.2% in our cohort is classified as “very low”, with 84% of participants in this category. The location of our cohort in the US Midwest region, as opposed to a coastal area with greater direct access to fresh seafood, may have contributed to the very low eO3I levels observed. Despite the low average levels, in the MILES Cohort, we found that eO3I as a continuous variable was favorably associated with patient-reported outcomes of SLE disease activity, pain, and sleep quality (**Figure 1**). Given the overall low mean eO3I levels, patients might further benefit by increasing their ω-3 PUFA intake via diet and supplement interventions.

### 4.8 Impact of increased intake of ω-3 PUFAs via diet and supplements on O3I

The ω-3 PUFA ALA, found in green plant tissues, plant oils (*e.g.*, canola, soybean, flax), and some nuts, can be elongated to EPA, DPA, and DHA, though conversion efficiency in humans is genetically variable (83) and often limited (84). These latter elongated ω-3 PUFAs can, however, be found in fatty fish, which when consumed twice weekly, is associated with reduced disease activity in RA (85). However, only 20 percent of Americans consume two servings of fish per week (86) and this dietary regimen is still insufficient to achieve a “desirable” range O3I of 8% or higher for most individuals (87, 88). Oily fish consumption is even more complicated in Michigan, where advisories recommend limiting consumption of Great Lakes trout and salmon to 6 to 12 servings per year due to contaminants such as polychlorinated biphenyls (PCBs) and methylmercury (89). We have previously reported that methylmercury exposure, even at levels generally deemed “safe” by regulatory agencies, is associated with high-titer antinuclear antibody positivity in reproductive-age women from the general population (90).

When ω-3 PUFA levels cannot be adequately increased through diet, supplements of fish oil containing DHA and EPA or microalgal oil, a vegan alternative containing DHA, can raise O3I levels from “low” to “desirable” (91). Walker et al. (92) analyzed data from 1422 individuals in 14 published ω-3 PUFA intervention trials, alongside baseline O3I values, to construct a regression model for predicting O3I responses to dosage. They determined the minimum doses of EPA + DHA necessary to attain a mean O3I of 8% within a 13-week period were 2.2 g/day starting from a baseline O3I of 2%, 1.5 from a 4% baseline, and 0.75 g from a 6% baseline. These daily doses are consistent with the European Food Safety Authority (93) tolerable daily upper intake level for EPA+DPA+DHA of 5 g/d. In our cohort, 82% of those in the very low O3I category did not use fish oil supplements, whereas the only two participants who attained the moderate O3I level reported regular supplement use.

### 4.9 Preclinical investigations linking dietary FAs to SLE manifestations

Preclinical studies in genetically prone and induced autoimmune mouse models have in general demonstrated preventive effects of ω-3 PUFA-enriched diets and exacerbation by diets high in ω-6 PUFAs, SFAs, and/or MUFAs (60, 73, 94–100). Consonant with these results, we found here that SFAs and MUFAs are associated with unfavorable SLE outcomes. However, in contrast to prior preclinical studies, we observed that when controlling for other types of fatty acids, serum ω-6 PUFAs, and specifically DGLA, were associated with better outcomes. It could be speculated that higher serum ω-6 PUFAs proportionally displace putatively more inflammatory SFAs and MUFAs, though more research is needed. Using the silica-triggered NZBWF1 autoimmune mouse model, feeding mice DHA at human equivalent doses of 2 to 5 g/d (which increased the O3I to >6%) was strongly linked to the suppression of autoimmune markers such as interferon-regulated gene expression, proinflammatory cytokine production, leukocyte infiltration, tertiary lymphoid tissue neogenesis, autoantibody production, and glomerulonephritis (100). In additional experiments using this mouse model to mimic gene-environment interaction in SLE, in mice fed a diet reflecting the average American diet, supplementation with DHA but not MUFAs delayed disease onset and suppressed the progression of the disease (74, 101, 102). Also in line with the identification here of DHA’s associations with beneficial effects, Halade et al. (60) found that DHA-enriched fish oil diets significantly prolonged both the median (658 days) and maximum (848 days) lifespan in lupus-prone NZBWF1 mice, while mice fed EPA-enriched fish oil had median and maximum lifespans of approximately 384 and 500 days, respectively. These investigators further reported that DHA diets were more efficacious than EPA diets at reducing dsDNA autoantibodies, glomerular IgG deposition, and proteinuria. Thus, DHA, rather than EPA, was the most effective ω-3 PUFA in suppressing kidney inflammation and extending the lifespan in this preclinical model of lupus. These findings are further consistent with our observations here that favorable associations with DHA were preeminent among the FAs analyzed.

### 4.10 Clinical effects of ω-3 PUFA supplementation on SLE

Akbar et al. (26) reviewed nine studies that examined the impact of fish oil supplementation on SLE or lupus nephritis, employing various study designs and durations ranging from 10 to 52 weeks and sample sizes ranging from 12 to 85 patients. Daily intake of ω-3 PUFAs ranged from 0.54 to 3.60 g EPA and 0.30 to 2.25 g DHA. Five of the seven SLE studies (n=27 to 62) showed significant improvements in disease activity (103–107), and one study (n=17) reported temporary improvement (108). In contrast, Bello and colleagues reported in a study of SLE patients (n=85) that daily consumption of 1.8 g EPA + 1.2 g DHA for 24 weeks did not impact clinical outcomes (109). A small study of SLE patients with and without lupus nephritis (LN) (n=12) using 1.1 to 3.2 g/d EPA and 0.7 to 2.2 g/d DHA for 10 weeks observed positive changes in several serum markers (98), whereas another study of lupus nephritis patients (n=52) who supplemented with 2.7 g/d EPA and 1.7 g/d DHA reported no significant improvements (110). Recently, in a randomized controlled trial of 78 persons with SLE, after four weeks of supplementation with 4 g/day krill oil (containing 722 mg EPA and 384 mg DHA), the O3I increased significantly from 4.4 to 7.2%, whereas there was no change in the placebo group (111). This study was powered to determine the impact on O3I. For the secondary endpoint of SLE activity [measured by the SLE Disease Activity Index 2000 (SLEDAI-2K)], overall, change in disease activity was similar between groups during the 24-week randomization period. However, in the subset of those with high disease activity (SLEDAI-2K ≥9) at baseline, there was a significant improvement in disease activity in the krill oil compared to placebo group at 4, 8, and 16 weeks. Altogether, while promising, insights gained from these clinical studies are hindered by low patient numbers, heterogeneous patient populations, type of ω-3 PUFA (mixture vs. purified), variable duration times, failure to determine ω-3 PUFA baseline or confirm post-intervention ω-3 PUFA increase, and not controlling for concomitant medications, infections, and disease activity.

### 4.11 Strengths and limitations

This study adds to the literature because it assesses numerous fatty acids, objectively measured by serum concentration as opposed to self-reported dietary intake histories. While dietary assessment via questionnaires captures habitual intake over specified periods of time, it is subject to recall biases and challenges in relating dietary habits to specific nutrient levels. A strength of the present study is that we were able to interrogate “internal doses” of individual fatty acids as well their major categories within serum total lipids, including nonesterified fatty acids, triacylglycerols, phospholipids, and cholesterol esters (112). Moreover, most clinical research on fatty acids in rheumatic diseases has focused on ω-3 PUFAs. Our ability to simultaneously interrogate multiple fatty acids provides valuable resolution on their relative effects. In addition, we utilized a variable selection approach (LASSO) that was agnostic to our hypotheses, adding credence to the favorable associations detected for DHA across all three outcome domains, which aligned with our expectations. Interestingly, unexpected antagonistic associations were observed between DHA and EPA, which warrant additional investigation. Since all fatty foods have a mix of fatty acid types, unexpected associations may serve as indicators of an overall unbalanced intake of fatty acids, and not necessarily due to absolute effects of any given food. Finally, given the population-based nature of the MILES Cohort, observations can be considered reasonably generalizable to persons with lupus in this region. This contrasts with studies confined to patients from tertiary care centers.

This study also has several limitations. First, due to the cross-sectional nature, temporal trends cannot be assessed and cannot exclude the possibility of reverse causality, whereby features of illness may impact dietary practices and supplement use (113). Second, while measurement of fatty acids in total serum lipids at a single time point was advantageous from the perspective of logistics and sample stability, levels can be subtly influenced by dietary intake timing, types and quantities of food/supplements consumed, fasting, and genetic polymorphisms in lipid synthesis, transport, and metabolism. Therefore, they may not reflect longer patterns of fatty acid intake to the same extent as the logistically more-difficult measurement in erythrocytes (112, 114). Third, dietary patterns and other characteristics of our US Midwest population may differ from those in other locales. Thus, extrapolation of results to other populations must be exercised with caution. A specific aspect for consideration is that our cohort as a whole was deficient in EPA and DHA, decreasing the likelihood of discriminating effects of increased consumption of these long chain PUFAs. In populations with higher baseline levels, it is possible that “dose responses” would differ. Fourth, unmeasured lifestyle factors, such as exercise, may correlate with healthy diets and contribute to beneficial outcomes. Fifth, although the demographics of our study population are reflective of southeastern Michigan, small demographic subsets in this region likewise are not well-represented in this study.

## 5 Conclusion

While causal links between fatty acid profiles and SLE outcomes are yet to be firmly established, strong evidence exists related to beneficial effects of increasing ω-3 PUFA status in cardiovascular and chronic kidney disease (115, 116), which are major causes of morbidity and mortality in SLE (2). Therefore, addressing fatty acid profiles in SLE may have broad health implications in this population. The vast majority (84%) of persons with SLE in this population-based cohort were estimated to have “very low” ω-3 PUFA tissue status. This deficient status could be improved considerably through precision nutrition addressing both dietary intake patterns and ω-3 PUFA supplementation (88). Whereas most SLE research on fatty acids has focused on ω-3 and ω-6 PUFAs, our findings suggest that modulating other types of fatty acids, such as SFAs and MUFAs, may also be important facets of a comprehensive dietary strategy in for SLE management. In the future, complementary investigation of circulating oxylipins in SLE could provide further perspectives on the mechanistic consequences of FA modulation in this autoimmune disease.

## Data Availability

Data supporting this study are not publicly available as these data are considered sensitive personal and health data and pertain to an uncommon disease in a defined geographic region. Requests to access the datasets should be directed to Dr. Emily Somers, emsomers@umich.edu.

## References

[1] Jorge A Wallace ZS Zhang Y Lu N Costenbader KH Choi HK. All-cause and cause-specific mortality trends of end-stage renal disease due to lupus nephritis from 1995 to 2014. Arthritis Rheumatol. (2019) 71:403–10. doi: 10.1002/art.2019.71.issue-3 PMC6393198 30225916

[2] Muñoz-Grajales C Yilmaz EB Svenungsson E Touma Z. Systemic lupus erythematosus and damage: What has changed over the past 20 years? Best Pract Res Clin Rheumatol. (2023) 37(4):101893. doi: 10.1016/j.berh.2023.101893 37993371

[3] Fanouriakis A Tziolos N Bertsias G Boumpas DT. Update on the diagnosis and management of systemic lupus erythematosus. Ann Rheum Dis. (2021) 80(1):14–25. doi: 10.1136/annrheumdis-2020-218272 33051219

[4] Sammaritano LR Bermas BL Chakravarty EE Chambers C Clowse MEB Lockshin MD. 2020 American College of Rheumatology Guideline for the management of reproductive health in rheumatic and musculoskeletal diseases. Arthritis Rheumatol (Hoboken NJ). (2020) 72:529–56. doi: 10.1002/art.41191 32090480

[5] Ruiz-Irastorza G Bertsias G. Treating systemic lupus erythematosus in the 21st century: new drugs and new perspectives on old drugs. Rheumatol (Oxford). (2020) 59:v69–81. doi: 10.1093/rheumatology/keaa403 PMC7719039 33280011

[6] Minhas D Marder W Harlow S Hassett AL Zick SM Gordon C. Access and cost-related nonadherence to prescription medications among lupus patients and controls: The Michigan Lupus Epidemiology and Surveillance Program. Arthritis Care Res (Hoboken). (2021) 73:1561–7. doi: 10.1002/acr.v73.11 PMC9219566 32741110

[7] Minhas D Marder W Hassett AL Zick SM Gordon C Harlow SD. Cost-related prescription non-adherence and patient-reported outcomes in systemic lupus erythematosus: The Michigan Lupus Epidemiology & Surveillance program. Lupus. (2023) 32:1075–83. doi: 10.1177/09612033231186113 PMC10585710 37378450

[8] Nicastro HL Vorkoper S Sterling R Korn AR Brown AGM Maruvada P. Opportunities to advance implementation science and nutrition research: a commentary on the Strategic Plan for NIH Nutrition Research. Transl Behav Med. (2023) 13:1–6. doi: 10.1093/tbm/ibac066 36370119 PMC10091491

[9] Robinson GA Mcdonnell T Wincup C Martin-Gutierrez L Wilton J Kalea AZ. Diet and lupus: what do the patients think? Lupus. (2019) 28:755–63. doi: 10.1177/0961203319845473 31027464

[10] Calder PC. Omega-3 fatty acids and inflammatory processes: from molecules to man. Biochem Soc Trans. (2017) 45:1105–15. doi: 10.1042/BST20160474 28900017

[11] Zeilhofer HU. Prostanoids in nociception and pain. Biochem Pharmacol. (2007) 73:165–74. doi: 10.1016/j.bcp.2006.07.037 16959219

[12] Korotkova M Jakobsson P-J. Persisting eicosanoid pathways in rheumatic diseases. Nat Rev Rheumatol. (2014) 10:229–41. doi: 10.1038/nrrheum.2014.1 24514915

[13] Sala A Proschak E Steinhilber D Rovati GE. Two-pronged approach to anti-inflammatory therapy through the modulation of the arachidonic acid cascade. Biochem Pharmacol. (2018) 158:161–73. doi: 10.1016/j.bcp.2018.10.007 30315753

[14] Wang B Wu L Chen J Dong L Chen C Wen Z. Metabolism pathways of arachidonic acids: mechanisms and potential therapeutic targets. Signal Transduct Target Ther. (2021) 6:94. doi: 10.1038/s41392-020-00443-w 33637672 PMC7910446

[15] Maroon JC Bost JW. Omega-3 fatty acids (fish oil) as an anti-inflammatory: an alternative to nonsteroidal anti-inflammatory drugs for discogenic pain. Surg Neurol. (2006) 65:326–31. doi: 10.1016/j.surneu.2005.10.023 16531187

[16] Ko GD Nowacki NB Arseneau L Eitel M Hum A. Omega-3 fatty acids for neuropathic pain: case series. Clin J Pain. (2010) 26:168–72. doi: 10.1097/AJP.0b013e3181bb8533 20090445

[17] Goldberg RJ Katz J. A meta-analysis of the analgesic effects of omega-3 polyunsaturated fatty acid supplementation for inflammatory joint pain. Pain. (2007) 129:210–23. doi: 10.1016/j.pain.2007.01.020 17335973

[18] Innes JK Calder PC. Omega-6 fatty acids and inflammation. Prostaglandins Leukot Essent Fatty Acids. (2018) 132:41–8. doi: 10.1016/j.plefa.2018.03.004 29610056

[19] Gioxari A Kaliora AC Marantidou F Panagiotakos DP. Intake of ω-3 polyunsaturated fatty acids in patients with rheumatoid arthritis: A systematic review and meta-analysis. Nutrition. (2018) 45:114–124.e4. doi: 10.1016/j.nut.2017.06.023 28965775

[20] Sigaux J Mathieu S Nguyen Y Sanchez P Letarouilly J-G Soubrier M. Impact of type and dose of oral polyunsaturated fatty acid supplementation on disease activity in inflammatory rheumatic diseases: a systematic literature review and meta-analysis. Arthritis Res Ther. (2022) 24:100. doi: 10.1186/s13075-022-02781-2 35526074 PMC9077862

[21] Gkiouras K Grammatikopoulou MG Myrogiannis I Papamitsou T Rigopoulou EI Sakkas LI. Efficacy of n-3 fatty acid supplementation on rheumatoid arthritis’ disease activity indicators: a systematic review and meta-analysis of randomized placebo-controlled trials. Crit Rev Food Sci Nutr. (2024) 64:16–30. doi: 10.1080/10408398.2022.2104210 35900212

[22] Matsumoto Y Sugioka Y Tada M Okano T Mamoto K Inui K. Monounsaturated fatty acids might be key factors in the Mediterranean diet that suppress rheumatoid arthritis disease activity: The TOMORROW study. Clin Nutr. (2018) 37:675–80. doi: 10.1016/j.clnu.2017.02.011 28285975

[23] Sekar S Panchal SK Ghattamaneni NK Brown L Crawford R Xiao Y. Dietary saturated fatty acids modulate pain behaviour in trauma-induced osteoarthritis in rats. Nutrients. (2020) 12(2):509. doi: 10.3390/nu12020509 32085385 PMC7071407

[24] Daien C Czernichow S Letarouilly J-G Nguyen Y Sanchez P Sigaux J. Dietary recommendations of the French Society for Rheumatology for patients with chronic inflammatory rheumatic diseases. Joint Bone Spine. (2022) 89:105319. doi: 10.1016/j.jbspin.2021.105319 34902577

[25] Maldonado-Pereira L Barnaba C de Los Campos G Medina-Meza IG. Evaluation of the nutritional quality of ultra-processed foods (ready to eat + fast food): Fatty acids, sugar, and sodium. J Food Sci. (2022) 87(8):3659–76. doi: 10.1111/1750-3841.16235 35781710

[26] Akbar U Yang M Kurian D Mohan C. Omega-3 fatty acids in rheumatic diseases: A critical review. J Clin Rheumatol. (2017) 23:330–9. doi: 10.1097/RHU.0000000000000563 28816722

[27] Charoenwoodhipong P Harlow SD Marder W Hassett AL McCune WJ Gordon C. Dietary omega polyunsaturated fatty acid intake and patient-reported outcomes in systemic lupus erythematosus: The Michigan Lupus Epidemiology and Surveillance Program. Arthritis Care Res (Hoboken). (2020) 72:874–81. doi: 10.1002/acr.23925 PMC6842394 31074595

[28] Vordenbäumen S Sokolowski A Kutzner L Rund KM Düsing C Chehab G. Erythrocyte membrane polyunsaturated fatty acid profiles are associated with systemic inflammation and fish consumption in systemic lupus erythematosus: a cross-sectional study. Lupus. (2020) 29:554–9. doi: 10.1177/0961203320912326 32188303

[29] Somers EC Marder W Cagnoli P Lewis EE DeGuire P Gordon C. Population-based incidence and prevalence of systemic lupus erythematosus: The Michigan Lupus Epidemiology and Surveillance Program. Arthritis Rheumatol. (2014) 66:369–78. doi: 10.1002/art.38238 PMC4198147 24504809

[30] Lim SS Drenkard C McCune WJ Helmick CG Gordon C DeGuire P. Population-based lupus registries: Advancing our epidemiologic understanding. Arthritis Care Res. (2009) 61(10):1462–6. doi: 10.1002/art.24835 19790117

[31] Centers for Disease Control and Prevention (CDC) National Center for Health Statistics (NCHS). National Health and Nutrition Examination Survey Demographic Questionnaire. Available online at: https://wwwn.cdc.gov/nchs/nhanes/. (Accessed December 2023).

[32] Karlson EW Daltroy LH Rivest C Ramsey-Goldman R Wright EA Partridge AJ. Validation of a Systemic Lupus Activity Questionnaire (SLAQ) for population studies. Lupus. (2003) 12:280–6. doi: 10.1191/0961203303lu332oa 12729051

[33] Ware JE Sherbourne CD. The MOS 36-item short-form health survey (SF-36). I. Conceptual framework and item selection. Med Care. (1992) 30:473–83. doi: 10.1097/00005650-199206000-00002 1593914

[34] NIH. PROMIS Scoring Manual - Sleep Disturbance (2015). Available online at: http://www.healthmeasures.net/administrator/components/com_instruments/uploads/15-09-01_15-11-24_PROMISSleepDisturbanceScoringManual.pdf. (Accessed April 2017)

[35] Lepage G Roy CC. Direct transesterification of all classes of lipids in a one-step reaction. J Lipid Res. (1986) 27:114–20. doi: 10.1016/S0022-2275(20)38861-1 3958609

[36] Masood A Stark KD Salem N. A simplified and efficient method for the analysis of fatty acid methyl esters suitable for large clinical studies. J Lipid Res. (2005) 46:2299–305. doi: 10.1194/jlr.D500022-JLR200 16061957

[37] Harris WS. The omega-3 index: from biomarker to risk marker to risk factor. Curr Atheroscler Rep. (2009) 11:411–7. doi: 10.1007/s11883-009-0062-2 19852881

[38] Chaudhary R Saadin K Bliden KP Harris WS Dinh B Sharma T. Risk factors associated with plasma omega-3 fatty acid levels in patients with suspected coronary artery disease. Prostaglandins Leukot Essent Fatty Acids. (2016) 113:40–5. doi: 10.1016/j.plefa.2016.08.009 27720039

[39] Buchanan CDC Lust CAC Burns JL Hillyer LM Martin SA Wittert GA. Analysis of major fatty acids from matched plasma and serum samples reveals highly comparable absolute and relative levels. Prostaglandins Leukot Essent Fatty Acids. (2021) 168:102268. doi: 10.1016/j.plefa.2021.102268 33831721

[40] Schuchardt JP Cerrato M Ceseri M DeFina LF Delgado GE Gellert S. Red blood cell fatty acid patterns from 7 countries: Focus on the Omega-3 index. Prostaglandins Leukot Essent Fat Acids. (2022) 179:102418. doi: 10.1016/j.plefa.2022.102418 PMC10440636 35366625

[41] Tibshirani R. Regression shrinkage and selection via the lasso. J R Stat Soc Ser B. (1996) 58:267–88. doi: 10.1111/j.2517-6161.1996.tb02080.x

[42] Okabe M Ito K. Color Universal Design (CUD) - How to make figures and presentations that are friendly to Colorblind people (2008). Available online at: https://jfly.uni-koeln.de/color/pallet.(Accessed April 2024).

[43] Gutiérrez S Svahn SL Johansson ME. Effects of omega-3 fatty acids on immune cells. Int J Mol Sci. (2019) 20(20):5028. doi: 10.3390/ijms20205028 31614433 PMC6834330

[44] Wong CK Wong PTY Tam LS Li EK Chen DP Lam CWK. Activation profile of Toll-like receptors of peripheral blood lymphocytes in patients with systemic lupus erythematosus. Clin Exp Immunol. (2010) 159:11–22. doi: 10.1111/j.1365-2249.2009.04036.x 19843090 PMC2802691

[45] Norris PC Dennis EA. A lipidomic perspective on inflammatory macrophage eicosanoid signaling. Adv Biol Regul. (2014) 54:99–110. doi: 10.1016/j.jbior.2013.09.009 24113376 PMC3946543

[46] Norris PC Gosselin D Reichart D Glass CK Dennis EA. Phospholipase A2 regulates eicosanoid class switching during inflammasome activation. Proc Natl Acad Sci USA. (2014) 111(35):12746–51. doi: 10.1073/pnas.1404372111 PMC4156727 25139986

[47] Favor OK Rajasinghe LD Wierenga KA Maddipati KR Lee KSS Olive AJ. Crystalline silica-induced proinflammatory eicosanoid storm in novel alveolar macrophage model quelled by docosahexaenoic acid supplementation. Front Immunol. (2023) 14:1274147. doi: 10.3389/fimmu.2023.1274147 38022527 PMC10665862

[48] Li X Yu Y Funk CD. Cyclooxygenase-2 induction in macrophages is modulated by docosahexaenoic acid via interactions with free fatty acid receptor 4 (FFA4). FASEB J. (2013) 27:4987–97. doi: 10.1096/fj.13-235333 24005906

[49] Yan Y Jiang W Spinetti T Tardivel A Castillo R Bourquin C. Omega-3 fatty acids prevent inflammation and metabolic disorder through inhibition of NLRP3 inflammasome activation. Immunity. (2013) 38:1154–63. doi: 10.1016/j.immuni.2013.05.015 23809162

[50] Serhan CN. Systems approach to inflammation resolution: identification of novel anti-inflammatory and pro-resolving mediators. J Thromb Haemost. (2009) 7 Suppl 1:44–8. doi: 10.1111/j.1538-7836.2009.03396.x 19630766

[51] Ostermann AI Schebb NH. Effects of omega-3 fatty acid supplementation on the pattern of oxylipins: a short review about the modulation of hydroxy-, dihydroxy-, and epoxy-fatty acids. Food Funct. (2017) 8:2355–67. doi: 10.1039/C7FO00403F 28682409

[52] Basil MC Levy BD. Specialized pro-resolving mediators: endogenous regulators of infection and inflammation. Nat Rev Immunol. (2016) 16:51–67. doi: 10.1038/nri.2015.4 26688348 PMC5242505

[53] Al-Shaer AE Buddenbaum N Shaikh SR. Polyunsaturated fatty acids, specialized pro-resolving mediators, and targeting inflammation resolution in the age of precision nutrition. Biochim Biophys Acta Mol Cell Biol Lipids. (2021) 1866:158936. doi: 10.1016/j.bbalip.2021.158936 33794384 PMC8496879

[54] Sham HP Walker KH Abdulnour R-EE Krishnamoorthy N Douda DN Norris PC. 15-epi-lipoxin A4, resolvin D2, and resolvin D3 induce NF-κB regulators in bacterial pneumonia. J Immunol. (2018) 200:2757–66. doi: 10.4049/jimmunol.1602090 PMC5906795 29523657

[55] Titos E Rius B López-Vicario C Alcaraz-Quiles J García-Alonso V Lopategi A. Signaling and immunoresolving actions of resolvin D1 in inflamed human visceral adipose tissue. J Immunol. (2016) 197:3360–70. doi: 10.4049/jimmunol.1502522 PMC5101161 27647830

[56] Chiang N Fredman G Bäckhed F Oh SF Vickery T Schmidt BA. Infection regulates pro-resolving mediators that lower antibiotic requirements. Nature. (2012) 484:524–8. doi: 10.1038/nature11042 PMC3340015 22538616

[57] Fredman G Hellmann J Proto JD Kuriakose G Colas RA Dorweiler B. An imbalance between specialized pro-resolving lipid mediators and pro-inflammatory leukotrienes promotes instability of atherosclerotic plaques. Nat Commun. (2016) 7:12859. doi: 10.1038/ncomms12859 27659679 PMC5036151

[58] So J Wu D Lichtenstein AH Tai AK Matthan NR Maddipati KR. EPA and DHA differentially modulate monocyte inflammatory response in subjects with chronic inflammation in part via plasma specialized pro-resolving lipid mediators: A randomized, double-blind, crossover study. Atherosclerosis. (2021) 316:90–8. doi: 10.1016/j.atherosclerosis.2020.11.018 33303222

[59] Wei MY Jacobson TA. Effects of eicosapentaenoic acid versus docosahexaenoic acid on serum lipids: a systematic review and meta-analysis. Curr Atheroscler Rep. (2011) 13:474–83. doi: 10.1007/s11883-011-0210-3 21975919

[60] Halade GV Rahman MM Bhattacharya A Barnes JL Chandrasekar B Fernandes G. Docosahexaenoic acid-enriched fish oil attenuates kidney disease and prolongs median and maximal life span of autoimmune lupus-prone mice. J Immunol. (2010) 184:5280–6. doi: 10.4049/jimmunol.0903282 PMC2952419 20368275

[61] Innes JK Calder PC. The differential effects of eicosapentaenoic acid and docosahexaenoic acid on cardiometabolic risk factors: a systematic review. Int J Mol Sci. (2018) 19(2):532. doi: 10.3390/ijms19020532 29425187 PMC5855754

[62] Allaire J Couture P Leclerc M Charest A Marin J Lépine M-C. A randomized, crossover, head-to-head comparison of eicosapentaenoic acid and docosahexaenoic acid supplementation to reduce inflammation markers in men and women: the Comparing EPA to DHA (ComparED) Study. Am J Clin Nutr. (2016) 104:280–7. doi: 10.3945/ajcn.116.131896 27281302

[63] Sergeant S Rahbar E Chilton FH. Gamma-linolenic acid, Dihommo-gamma linolenic, Eicosanoids and Inflammatory Processes. Eur J Pharmacol. (2016) 785:77–86. doi: 10.1016/j.ejphar.2016.04.020 27083549 PMC4975646

[64] Baker EJ Valenzuela CA van Dooremalen WTM Martínez-Fernández L Yaqoob P Miles EA. Gamma-linolenic and pinolenic acids exert anti-inflammatory effects in cultured human endothelial cells through their elongation products. Mol Nutr Food Res. (2020) 64(20):e2000382. doi: 10.1002/mnfr.202000382 32898315

[65] Ouchi S Miyazaki T Shimada K Sugita Y Shimizu M Murata A. Decreased circulating dihomo-gamma-linolenic acid levels are associated with total mortality in patients with acute cardiovascular disease and acute decompensated heart failure. Lipids Health Dis. (2017) 16:150. doi: 10.1186/s12944-017-0542-2 28806965 PMC5556673

[66] Elkan A-C Anania C Gustafsson T Jogestrand T Hafström I Frostegård J. Diet and fatty acid pattern among patients with SLE: associations with disease activity, blood lipids and atherosclerosis. Lupus. (2012) 21:1405–11. doi: 10.1177/0961203312458471 22930204

[67] Gorczyca D Szponar B Paściak M Czajkowska A Szmyrka M. Serum levels of n-3 and n-6 polyunsaturated fatty acids in patients with systemic lupus erythematosus and their association with disease activity: a pilot study. Scand J Rheumatol. (2022) 51:230–6. doi: 10.1080/03009742.2021.1923183 34169789

[68] Osthues T Sisignano M. Oxidized lipids in persistent pain states. Front Pharmacol. (2019) 10:1147. doi: 10.3389/fphar.2019.01147 31680947 PMC6803483

[69] Ji R-R. Specialized pro-resolving mediators as resolution pharmacology for the control of pain and itch. Annu Rev Pharmacol Toxicol. (2023) 63:273–93. doi: 10.1146/annurev-pharmtox-051921-084047 PMC10290889 36100219

[70] Kawashima H Yoshizawa K. The physiological and pathological properties of Mead acid, an endogenous multifunctional n-9 polyunsaturated fatty acid. Lipids Health Dis. (2023) 22:172. doi: 10.1186/s12944-023-01937-6 37838679 PMC10576882

[71] Patel P Cossette C Anumolu JR Gravel S Lesimple A Mamer OA. Structural requirements for activation of the 5-oxo-6E,8Z, 11Z,14Z-eicosatetraenoic acid (5-oxo-ETE) receptor: identification of a mead acid metabolite with potent agonist activity. J Pharmacol Exp Ther. (2008) 325:698–707. doi: 10.1124/jpet.107.134908 18292294

[72] Powell WS Rokach J. The eosinophil chemoattractant 5-oxo-ETE and the OXE receptor. Prog Lipid Res. (2013) 52:651–65. doi: 10.1016/j.plipres.2013.09.001 PMC5710732 24056189

[73] Alexander NJ Smythe NL Jokinen MP. The type of dietary fat affects the severity of autoimmune disease in NZB/NZW mice. Am J Pathol. (1987) 127:106–21. PMC1899609 3565532

[74] Gilley KN Wierenga KA Chauhuan PS Wagner JG Lewandowski RP Ross EA. Influence of total western diet on docosahexaenoic acid suppression of silica-triggered lupus flaring in NZBWF1 mice. PloS One. (2020) 15:e0233183. doi: 10.1371/journal.pone.0233183 32413078 PMC7228097

[75] Tierney JA Uong CD Lenert ME Williams M Burton MD. High-fat diet causes mechanical allodynia in the absence of injury or diabetic pathology. Sci Rep. (2022) 12:14840. doi: 10.1038/s41598-022-18281-x 36050326 PMC9437006

[76] Galán-Arriero I Serrano-Muñoz D Gómez-Soriano J Goicoechea C Taylor J Velasco A. The role of omega-3 and omega-9 fatty acids for the treatment of neuropathic pain after neurotrauma. Biochim Biophys Acta Biomembr. (2017) 1859:1629–35. doi: 10.1016/j.bbamem.2017.05.003 28495596

[77] Patan MJ Kennedy DO Husberg C Hustvedt SO Calder PC Middleton B. Differential effects of DHA- and EPA-rich oils on sleep in healthy young adults: a randomized controlled trial. Nutrients. (2021) 13(1):248. doi: 10.3390/nu13010248 33467135 PMC7830450

[78] Yokoi-Shimizu K Yanagimoto K Hayamizu K. Effect of docosahexaenoic acid and eicosapentaenoic acid supplementation on sleep quality in healthy subjects: a randomized, double-blinded, placebo-controlled trial. Nutrients. (2022) 14(19):4136. doi: 10.3390/nu14194136 36235788 PMC9573173

[79] Murphy RA Devarshi PP Mun JG Marshall K Mitmesser SH. Association of omega-3 levels and sleep in US adults, National Health and Nutrition Examination Survey, 2011-2012. Sleep Health. (2022) 8:294–7. doi: 10.1016/j.sleh.2021.12.003 35153167

[80] Murphy RA Tintle N Harris WS Darvishian M Marklund M Virtanen JK. PUFA ω-3 and ω-6 biomarkers and sleep: a pooled analysis of cohort studies on behalf of the Fatty Acids and Outcomes Research Consortium (FORCE). Am J Clin Nutr. (2022) 115:864–76. doi: 10.1093/ajcn/nqab408 PMC8895226 34918026

[81] Peuhkuri K Sihvola N Korpela R. Diet promotes sleep duration and quality. Nutr Res. (2012) 32:309–19. doi: 10.1016/j.nutres.2012.03.009 22652369

[82] Stark KD Van Elswyk ME Higgins MR Weatherford CA Salem N. Global survey of the omega-3 fatty acids, docosahexaenoic acid and eicosapentaenoic acid in the blood stream of healthy adults. Prog Lipid Res. (2016) 63:132–52. doi: 10.1016/j.plipres.2016.05.001 27216485

[83] Smith CE Follis JL Nettleton JA Foy M Wu JHY Ma Y. Dietary fatty acids modulate associations between genetic variants and circulating fatty acids in plasma and erythrocyte membranes: Meta-analysis of nine studies in the CHARGE consortium. Mol Nutr Food Res. (2015) 59:1373–83. doi: 10.1002/mnfr.201400734 PMC4491005 25626431

[84] Baker EJ Miles EA Burdge GC Yaqoob P Calder PC. Metabolism and functional effects of plant-derived omega-3 fatty acids in humans. Prog Lipid Res. (2016) 64:30–56. doi: 10.1016/j.plipres.2016.07.002 27496755

[85] Tedeschi SK Bathon JM Giles JT Lin T-C Yoshida K Solomon DH. Relationship between fish consumption and disease activity in rheumatoid arthritis. Arthritis Care Res (Hoboken). (2018) 70:327–32. doi: 10.1002/acr.23295 PMC5740014 28635117

[86] Terry A Herrick K Afful J Ahluwalia N. Seafood Consumption in the United States, 2013–2016 NCHS Data Brief no 321. Hyattsville, MD: National Center for Health Statistics (2018). 30312150

[87] McDonnell SL French CB Baggerly CA Harris WS. Cross-sectional study of the combined associations of dietary and supplemental eicosapentaenoic acid + docosahexaenoic acid on Omega-3 Index. Nutr Res. (2019) 71:43–55. doi: 10.1016/j.nutres.2019.09.001 31757628

[88] Jackson KH Polreis JM Tintle NL Kris-Etherton PM Harris WS. Association of reported fish intake and supplementation status with the omega-3 index. Prostaglandins Leukot Essent Fatty Acids. (2019) 142:4–10. doi: 10.1016/j.plefa.2019.01.002 30773210

[89] Cleary BM Romano ME Chen CY Heiger-Bernays W Crawford KA. Comparison of recreational fish consumption advisories across the USA. Curr Environ Health Rep. (2021) 8:71–88. doi: 10.1007/s40572-021-00312-w 33934293 PMC8208921

[90] Somers EC Ganser MA Warren JS Basu N Wang L Zick SM. Mercury exposure and antinuclear antibodies among females of reproductive age in the United States: NHANES. Environ Health Perspect. (2015) 123:792–8. doi: 10.1289/ehp.1408751 PMC4529012 25665152

[91] Dempsey M Rockwell MS Wentz LM. The influence of dietary and supplemental omega-3 fatty acids on the omega-3 index: A scoping review. Front Nutr. (2023) 10:1072653. doi: 10.3389/fnut.2023.1072653 36742439 PMC9892774

[92] Walker RE Jackson KH Tintle NL Shearer GC Bernasconi A Masson S. Predicting the effects of supplemental EPA and DHA on the omega-3 index. Am J Clin Nutr. (2019) 110:1034–40. doi: 10.1093/ajcn/nqz161 31396625

[93] European Food Safety Authority (EFSA). Scientific opinion on the tolerable upper intake level of eicosapentaenoic acid (EPA), docosahexaenoic acid (DHA) and docosapentaenoic acid (DPA). EFSA J. (2012) 10(7):2815. doi: 10.2903/j.efsa.2012.2815 PMC13093066 42016086

[94] Westberg G Tarkowski A Svalander C. Effect of eicosapentaenoic acid rich menhaden oil and MaxEPA on the autoimmune disease of Mrl/l mice. Int Arch Allergy Appl Immunol. (1989) 88:454–61. doi: 10.1159/000234732 2542168

[95] Halade GV Williams PJ Veigas JM Barnes JL Fernandes G. Concentrated fish oil (Lovaza(R)) extends lifespan and attenuates kidney disease in lupus-prone short-lived (NZBxNZW)F1 mice. Exp Biol Med (Maywood). (2013) 238:610–22. doi: 10.1177/1535370213489485 PMC3970264 23918873

[96] Pestka JJ Vines LL Bates MA He K Langohr I. Comparative effects of n-3, n-6 and n-9 unsaturated fatty acid-rich diet consumption on lupus nephritis, autoantibody production and CD4+ T cell-related gene responses in the autoimmune NZBWF1 mouse. PloS One. (2014) 9:e100255. doi: 10.1371/journal.pone.0100255 24945254 PMC4063768

[97] Prickett JD Robinson DR Steinberg AD. A diet enriched with eicosapentaenoic acid suppresses autoimmune nephritis in female (NZB x NZW) F1 mice. Trans Assoc Am Physicians. (1982) 95:145–54. 6304973

[98] Clark WF Parbtani A Huff MW Reid B Holub BJ Falardeau P. Omega-3 fatty acid dietary supplementation in systemic lupus erythematosus. Kidney Int. (1989) 36:653–60. doi: 10.1038/ki.1989.242 2811063

[99] Chandrasekar B Troyer DA Venkatraman JT Fernandes G. Dietary omega-3 lipids delay the onset and progression of autoimmune lupus nephritis by inhibiting transforming growth factor beta mRNA and protein expression. J Autoimmun. (1995) 8:381–93. doi: 10.1006/jaut.1995.0030 7575999

[100] Wierenga KA Strakovsky RS Benninghoff AD Rajasinghe LD Lock AL Harkema JR. Requisite omega-3 HUFA biomarker thresholds for preventing murine lupus flaring. Front Immunol. (2020) 11:1796. doi: 10.3389/fimmu.2020.01796 32973753 PMC7473030

[101] Bates MA Akbari P Gilley KN Wagner JG Li N Kopec AK. Dietary docosahexaenoic acid prevents silica-induced development of pulmonary ectopic germinal centers and glomerulonephritis in the lupus-prone NZBWF1 mouse. Front Immunol. (2018) 9:2002. doi: 10.3389/fimmu.2018.02002 30258439 PMC6143671

[102] Benninghoff AD Bates MA Chauhan PS Wierenga KA Gilley KN Holian A. Docosahexaenoic acid consumption impedes early interferon- and chemokine-related gene expression while suppressing silica-triggered flaring of murine lupus. Front Immunol. (2019) 10:2851. doi: 10.3389/fimmu.2019.02851 31921124 PMC6923248

[103] Arriens C Hynan LS Lerman RH Karp DR Mohan C. Placebo-controlled randomized clinical trial of fish oil’s impact on fatigue, quality of life, and disease activity in systemic lupus erythematosus. Nutr J. (2015) 14:82. doi: 10.1186/s12937-015-0068-2 26283629 PMC4538741

[104] Lozovoy MAB Simão ANC Morimoto HK Scavuzzi BM Iriyoda TVM Reiche EMV. Fish oil N-3 fatty acids increase adiponectin and decrease leptin levels in patients with systemic lupus erythematosus. Mar Drugs. (2015) 13:1071–83. doi: 10.3390/md13021071 PMC4344620 25690094

[105] Wright SA O’Prey FM McHenry MT Leahey WJ Devine AB Duffy EM. A randomised interventional trial of omega-3-polyunsaturated fatty acids on endothelial function and disease activity in systemic lupus erythematosus. Ann Rheum Dis. (2008) 67:841–8. doi: 10.1136/ard.2007.077156 17875549

[106] Duffy EM Meenagh GK McMillan SA Strain JJ Hannigan BM Bell ALJL. The clinical effect of dietary supplementation with omega-3 fish oils and/or copper in systemic lupus erythematosus. J Rheumatol. (2004) 31:1551–6. 15290734

[107] Walton AJ Snaith ML Locniskar M Cumberland AG Morrow WJ Isenberg DA. Dietary fish oil and the severity of symptoms in patients with systemic lupus erythematosus. Ann Rheum Dis. (1991) 50:463–6. doi: 10.1136/ard.50.7.463 PMC1004457 1877851

[108] Westberg G Tarkowski A. Effect of MaxEPA in patients with SLE. A double-blind, crossover study. Scand J Rheumatol. (1990) 19(2):137–43. doi: 10.3109/03009749009102117 2186476

[109] Bello KJ Fang H Fazeli P Bolad W Corretti M Magder LS. Omega-3 in SLE: a double-blind, placebo-controlled randomized clinical trial of endothelial dysfunction and disease activity in systemic lupus erythematosus. Rheumatol Int. (2013) 33:2789–96. doi: 10.1007/s00296-013-2811-3 PMC3805738 23817872

[110] Clark WF Parbtani A. Omega-3 fatty acid supplementation in clinical and experimental lupus nephritis. Am J Kidney Dis. (1994) 23:644–7. doi: 10.1016/S0272-6386(12)70273-1 8172205

[111] Salmon J Wallace DJ Rus V Cox A Dykas C Williams B. Correction of omega-3 fatty acid deficiency and improvement in disease activity in patients with systemic lupus erythematosus treated with krill oil concentrate: a multicentre, randomised, double-blind, placebo-controlled trial. Lupus Sci Med. (2024) 11(2):e001201. doi: 10.1136/lupus-2024-001201 39009356 PMC11268053

[112] Brenna JT Plourde M Stark KD Jones PJ Lin Y-H. Best practices for the design, laboratory analysis, and reporting of trials involving fatty acids. Am J Clin Nutr. (2018) 108:211–27. doi: 10.1093/ajcn/nqy089 PMC6084616 29931035

[113] Oh J Oda K Brash M Beeson WL Sabaté J Fraser GE. Systemic lupus erythematosus and the ratio of omega-3 to omega-6 fatty acids consumption among women in the Adventist Health Study-2. Lupus. (2023) 32:1637–45. doi: 10.1177/09612033231213145 PMC10873066 37927031

[114] Harris WS Thomas RM. Biological variability of blood omega-3 biomarkers. Clin Biochem. (2010) 43:338–40. doi: 10.1016/j.clinbiochem.2009.08.016 19733159

[115] Hu Y Hu FB Manson JE. Marine omega-3 supplementation and cardiovascular disease: an updated meta-analysis of 13 randomized controlled trials involving 127 477 participants. J Am Heart Assoc. (2019) 8:e013543. doi: 10.1161/JAHA.119.013543 31567003 PMC6806028

[116] Ong KL Marklund M Huang L Rye K-A Hui N Pan X-F. Association of omega 3 polyunsaturated fatty acids with incident chronic kidney disease: pooled analysis of 19 cohorts. BMJ. (2023) 380:e072909. doi: 10.1136/bmj-2022-072909 36653033 PMC9846698

